# Green synthesis of magnetite iron oxide nanoparticles using *Azadirachta indica* leaf extract loaded on reduced graphene oxide and degradation of methylene blue

**DOI:** 10.1038/s41598-024-69184-y

**Published:** 2024-08-06

**Authors:** Muhammad Shahbaz Akhtar, Sania Fiaz, Sohaib Aslam, Shinho Chung, Allah Ditta, Muhammad Atif Irshad, Amal M. Al-Mohaimeed, Rashid Iqbal, Wedad A. Al-onazi, Muhammad Rizwan, Yoshitaka Nakashima

**Affiliations:** 1 Department of Environmental Sciences, Forman Christian College University, Lahore, 54600 Pakistan; 2 Department of Environmental Sciences, Shaheed Benazir Bhutto University, Sheringal, Dir (U), 18000 Pakistan; 3 School of Biological Sciences, The University of Western Australia, 35 Stirling Highway, Perth, WA 6009 Australia; 4 Department of Environmental Sciences, University of Lahore, Lahore, 54000 Pakistan; 5 Department of Chemistry, College of Science, King Saud University, P.O. Box 22452, 11495 Riyadh, Saudi Arabia; 6 Department of Agronomy, Faculty of Agriculture and Environment, The Islamia University of Bahawalpur, Bahawalpur, 63100 Pakistan; 7 Department of Life Sciences, Western Caspian University, Baku, Azerbaijan; 8 Institute of Crop Science and Resource Conservation (INRES), University of Bonn, 53115 Bonn, Germany; 9 Graduate School of Environmental, Life, Natural Science and Technology, Okayama University, Okayama, Japan

**Keywords:** *Azadirachta indica*, Graphene-based nanomaterials, Green deposition, Reduced graphene oxide, Nanocomposite, Methylene blue, Environmental sciences, Biomaterials

## Abstract

In the current arena, new-generation functional nanomaterials are the key players for smart solutions and applications including environmental decontamination of pollutants. Among the plethora of new-generation nanomaterials, graphene-based nanomaterials and nanocomposites are in the driving seat surpassing their counterparts due to their unique physicochemical characteristics and superior surface chemistry. The purpose of the present research was to synthesize and characterize magnetite iron oxide/reduced graphene oxide nanocomposites (FeNPs/rGO) via a green approach and test its application in the degradation of methylene blue. The modified Hummer's protocol was adopted to synthesize graphene oxide (GO) through a chemical exfoliation approach using a graphitic route. Leaf extract of *Azadirachta indica* was used as a green reducing agent to reduce GO into reduced graphene oxide (rGO). Then, using the green deposition approach and *Azadirachta indica* leaf extract, a nanocomposite comprising magnetite iron oxides and reduced graphene oxide i.e., FeNPs/rGO was synthesized. During the synthesis of functionalized FeNPs/rGO, *Azadirachta indica* leaf extract acted as a reducing, capping, and stabilizing agent. The final synthesized materials were characterized and analyzed using an array of techniques such as scanning electron microscopy (SEM)-energy dispersive X-ray microanalysis (EDX), Fourier transform infrared spectroscopy (FT-IR), X-ray diffraction analysis, and UV–visible spectrophotometry. The UV–visible spectrum was used to evaluate the optical characteristics and band gap. Using the FT-IR spectrum, functional groupings were identified in the synthesized graphene-based nanomaterials and nanocomposites. The morphology and elemental analysis of nanomaterials and nanocomposites synthesized via the green deposition process were investigated using SEM–EDX. The GO, rGO, FeNPs, and FeNPs/rGO showed maximum absorption at 232, 265, 395, and 405 nm, respectively. FTIR spectrum showed different functional groups (OH, COOH, C=O), C–O–C) modifying material surfaces. Based on Debye Sherrer's equation, the mean calculated particle size of all synthesized materials was < 100 nm (GO = 60–80, rGO = 90–95, FeNPs = 70–90, Fe/GO = 40–60, and Fe/rGO = 80–85 nm). Graphene-based nanomaterials displayed rough surfaces with clustered and spherical shapes and EDX analysis confirmed the presence of both iron and oxygen in all the nanocomposites. The final nanocomposites produced via the synthetic process degraded approximately 74% of methylene blue. Based on the results, it is plausible to conclude that synthesized FeNPs/rGO nanocomposites can also be used as a potential photocatalyst degrader for other different dye pollutants due to their lower band gap.

## Introduction

Global freshwater resources are limited and declining day by day. Water quality is challenged due to point and non-point sources of pollution. Freshwater resources are contaminated with industrial discharges/effluents and domestic wastes with nuisance organic and inorganic pollutants such as polyaromatic hydrocarbons (PAHs), persistent organic compounds (POPs), dyes, metals, and metalloids^1,2^. For safe and clean water, noxious pollutants should be removed from water by economically viable and efficient approaches. Rapid population expansion, increasing industrialization, urbanization, and pervasive agricultural practices have produced wastewater, which has rendered the water not just polluted but also toxic. On a global scale, millions of people die each year from illnesses caused by the consumption of water contaminated with dangerous microorganisms and toxic pollutants^3^. The treatment of wastewater has become a real global challenge. The requirement for chemicals, the synthesis of disinfection byproducts, the length of the process, and economics are the limiting factors for the use of different wastewater treatment techniques that have been reported over the last few decades^4^.

Aquatic ecosystems are being contaminated with dyes used in the textile industry. Approximately, 15% of globally produced dyes end up as toxic effluents and contaminants^5^. During textile chemical processes, artificial dyes such as azo dyes undergo rapid decomposition and become toxic to the environment due to the formation of aromatic amines because of double bonds in nitrogen^6^. Thus, effluents discharged from industrial chemical processes result in the coloration of water and the addition of toxic/hazardous substances that are a real threat to aquatic ecosystems^7,8^. Therefore, International Environmental Standards (IES) have been imposed to create awareness among the public about the threats of effluents released from industry^8^. Different physicochemical and biological approaches are reported in pertinent literature for the removal of pollutants e.g., dyes from the wastewater^9,10^. Conventional methods such as flocculation/coagulation, electrochemical and advanced oxidative processes, activated sludge process, reverse osmosis technique, and sorption are thoroughly investigated for the removal of pollutants^11–17^. Recently, the photocatalytic degradation technique emerged as a promising approach by using catalysts and light sources. In this technique, the generation of photo-induced OH radicals accelerates the photodegradation of organic effluents into non-toxic chemical species such as water and carbon dioxide without reliance on any separation technique^18–23^. The treated wastewater can be reused in agricultural, chemical, and textile sectors.

These days, nanotechnology is emerging as a powerful interdisciplinary tool and gaining much interest because of its performance and efficacy. Its extraordinary ability to build new atomic structures has already sparked the development of cutting-edge tools and materials with a diverse variety of applications^24^. Nowadays, multifunctional next-generation nanomaterials and nanocomposites are being synthesized by exploiting the physicochemical characteristics of nanostructures. These physicochemical properties include low density, high adsorptive surface area, more functional groups, differential shapes/orientations, and chemical fitness and suitability^25,26^. Along with other diverse applications, these nanostructures can treat water/wastewater. Because of their high surface-to-volume ratio, high sensitivity and reactivity, high adsorption capacity, and ease of functionalization, nanomaterials and nanocomposites are particularly well suited for application in wastewater treatment. Different wastewater treatment techniques include adsorption/biosorption, nanofiltration, photocatalysis, disinfection, and sensor technologies^27^. More recently, the use of nanocomposite photocatalysts is gaining much interest as a cutting-edge technique for the breakdown of pollutants present in wastewater. This technology has shown tremendous potential to treat wastewater since it uses nanocomposite photocatalysts made of doped graphene^28^.

The superior and wonderful substance known as graphene (G) has a two-dimensional (2D) hexagonal honeycomb lattice structure with a benzene (C_6_H_6_) ring. Pristine graphene is referred to be a semi-metal or zero-bandgap material since there is no energy difference between its valence and conduction bands. Graphene can be synthesized by two major routes i.e., bottom-up and top-down routes^29^. The discovery of graphene has had a profound impact on different scientific, engineering, agricultural, medical, and environmental disciplines, particularly after the investigation of Novoselov et al.^30^. Graphene is characterized by exceptional traits such as high electro-thermal conductivity, low density, and excellent mechanical, carrier, and optical properties^31–33^. To improve the solubility, conductivity, and physicochemical properties of G, functionalization (covalent or non-covalent) and fabrication of G are done to synthesize modified graphene-based materials such as graphene oxide (GO), reduced GO, G-based derivates, and nanocomposites. These G-based nanomaterials/nanocomposites are superior in morphological and physicochemical characteristics^25,26,34,35^.

At the advent of the Industrial Revolution, point and non-point releases of industrial effluents and hazardous wastes have increased environmental risks. Both colored and non-colored industrial wastewater effluents have detrimental effects on human health because of the presence of different pollutants. Several methods, such as adsorption onto adsorbents like activated carbon, flocculation, chemical oxidation, ultrafiltration, and clays are used to treat industrial wastewater. Due to the high cost of these technologies, photocatalysis is considered one of the most straightforward and ecologically benign methods for the treatment of wastewater. The key advantage of this approach is that water pollution is broken down using solar energy and a straightforward laboratory setup^36^.

Recently, a greener approach has been developed to synthesize nanoparticles and nanocomposites by using green chemistry. Conventional methods for synthesizing nanoparticles have a variety of negative effects on the environment and human life. When synthesizing nanoparticles/nanocomposites with traditional methods, toxic/hazardous chemicals and high temperatures are frequently used. To meet this challenge, concepts of green chemistry are manipulated in science and engineering that have resulted in different eco-friendly fields such as green nanotechnology. This approach helps in establishing green processes that are clean, safe, and environment friendly that can replace in practice chemical and physical processes producing nanomaterials and nanocomposites. More recently, green synthesis has been gaining much interest because it is eco-friendly, cost-effective, natural, renewable, and safe with minimal generation of toxic/hazardous substances and uses no or less strong oxidizing or reducing^37–40^. Plant-based extracts are enriched with different compounds and substances such as terpenoids and phenolic compounds that can be deployed for the reduction of metallic salts to nanoparticles and can hinder nanoparticle aggregation due to capping or stabilizing characteristics^41,42^. *Azadirachta indica* used in the present investigation is enriched in diverse phytochemicals and compounds such as carotenoids, terpenoids, triterpenoid (nimbin), flavonoids, glycosides, alkaloids, salannin, tannin and phenolic substances^37,43–45^. Phytochemicals derived from *Azadirachta indica* have the potential to sorb on metallic nanoparticle surfaces in addition to their capping property. Furthermore, reducing sugars in the extract of *Azadirachta indica* is capable of reducing metallic ions and can form metallic nanoparticles^46–48^. Natural products and their derivatives, including wine, different amino acids, glucose, and plant extracts, all include polyphenols, which function as reducing, capping, and stabilizing agents. Similarly, microorganisms including bacteria, yeast, and algae can also be used for the ecologically benign synthesis of nanoparticles^49^. The present study was conducted to synthesize and characterize iron oxide/reduced graphene oxide (FeNPs/rGO) nanocomposites synthesized via the green approach and tested its application in photocatalytic degradation of methylene blue.

## Materials and methods

Fresh leaves of *Azadirachta indica* were taken from the botanical garden of Forman Christian College University, Lahore (31°32′58.9′′ N 74°20′37.0′′ E). Reagent grade Iron (III) chloride hexahydrate (FeCl_3_·6H_2_O), methylene blue, sodium hydroxide (NaOH), graphite powder, concentrated sulfuric acid (H_2_SO_4_), potassium permanganate (KMnO4), hydrogen peroxide (H_2_O_2_), and hydrochloric acid (HCl) were obtained from Sigma-Aldrich and utilized without additional purification.

### Preparation of leaf extract

The branches of *Azadirachta indica* were stripped of their leaves after collection from the botanical garden of Forman Christian College, University, Lahore. To remove the dirt and debris, collected leaves were washed with tap water (once) and distilled water (twice). After washing, the leaves were air-dried for a week in the shade. Using an electronic grinder, dried leaves of *Azadirachta indica* were ground into a fine powder, which was then sieved through a fine mesh sieve. In a 500 mL beaker, 1.35 g of plant powder and 200 mL of distilled water were added. On a hot plate, stirring was done continuously for two hours in a beaker. The plant extract was filtered using Whatman no. 40 filter paper after two hours of stirring. After filtration, 160 mL of brown-colored plant extract was collected. This was in line with the method reported by Bhuiyan et al.^50^.

### Synthesis of graphene oxide

One gram of graphite powder and 50 mL of concentrated H_2_SO_4_ were added to a 500 mL beaker and agitated for about 30 min to synthesize graphene oxide (GO) using a modified Hummer technique. The graphite powder gave the solution its dark color. For one hour, the mixture was cooled to below 5 °C. Six grams of KMnO_4_ were added to the reaction mixture and then continuously agitated for two hours at a temperature below 15 °C. Then, 90 mL of distilled water was added dropwise to start the oxidation process. After the reaction, the liquid turned dark brown, and the temperature was constantly maintained below 30 °C and stirred for two hours. Then, 280 mL of distilled water and 6 mL of H_2_O_2_ were added to terminate the reaction and to remove any extra KMnO_4_. When the solution was forcefully swirled, a brilliant yellow hue resulted from the reaction. The reaction mixture was filtered before being cleaned with 10% HCl and distilled water. The final product was oven-dried for eight hours at 80 °C. The resultant GO displayed a similar appearance and properties as reported by Ishtiaq et al.^51^.

### Green synthesis of reduced graphene oxide using plant extract

Reduced graphene oxide (rGO) was synthesized via a green approach by using *Azadirachta indica* plant extract as a capping agent. One gram of synthetic graphene and 5 g of plant extract were combined in a 500 mL beaker, and the mixture was then continuously swirled at 70 °C for an hour to accomplish full reduction. Centrifuged after full reduction, and then rinsed with distilled water and ethanol to achieve pH neutrality following an oven drying process at 80 °C for about 8 h. The method was followed as reported by Anwar et al.^52^.

### Synthesis of magnetite iron nanoparticles

Ferric chloride hexahydrate (FeCl_3_.6H_2_O) was used as a precursor material to synthesize magnetite Fe_2_O_3_ nanoparticles. At room temperature, a 1:1 mixture of 50 mL of *Azadirachta indica* plant leaf extract and 50 mL of 0.1 M FeCl_3_.6H_2_O solution was added dropwise. Then, 1 M of NaOH was added till the pH reached 11. The production of an intensely black-colored solution after stirring the resulting mixture for about 30 min with a magnetic stirrer demonstrated the synthesis of iron oxide nanoparticles (FeNPs). Centrifugation of the synthesized nanoparticles at 4500 rpm was done for 15 min and washed thrice with ethanol and distilled water and separated these FeNPs. The FeNPs were dried in an oven for 24 h at 65 °C. After drying, magnetite iron nanoparticles were obtained, and their synthesis was in line with Bhuiyan et al.^50^.

### Synthesis of magnetite iron oxides/reduced graphene oxide nanocomposites using *Azadirachta indica* plant leaf extract

Green deposition was used for producing iron oxide/reduced graphene oxide (FeNPs/rGO) nanocomposites. Iron oxide and reduced graphene oxide were used at a weight ratio of 1:2. Iron oxides (0.08 g) and reduced graphene oxide (0.16 g) were collected individually in 100 mL beakers with 10 mL of distilled water. Both solution combinations were sonicated in an ultrasonicator for 30 min to improve suspension. For three hours, a solution of reduced graphene oxide was stirred continuously on a hot plate while a suspension of iron oxides was fed into it at a rate of around 0.5 mL every ten minutes. Following that, the solution was centrifuged at 4500 rpm for 10 min, and washing was carried out in three phases using distilled water and ethanol to remove any undesirable contaminants. The product was then heated up for 8 h at 65 °C to dry it. The dried product was ground into a fine powder containing magnetite iron oxides and reduced graphene oxide nanocomposite (FeNPs/rGO).

### Characterization techniques

For optical evaluations, the absorption spectra of synthesized nanocomposites were analyzed using UV–visible spectroscopy (Cary 50) in the 200–800 nm range. Agilent technology (Cary 630) FT-IR in the 650–4000/cm range was used to investigate functional groups. The crystal phase composition was studied using X-ray diffraction (Bruker D2 Phaser) in the 2θ range 10–80°. The mean particle size of all the synthesized nanomaterials by employing the deby Sherer’s equation. Morphology and elemental analyses were conducted using SEM–EDX (S-3400 N), Hitachi.

### Photocatalytic activity

The photocatalytic activity of each sample was assessed against methylene blue (MB) with and without the presence of the catalyst. A 100 mL beaker was filled with 25 ml of a 20 ppm MB solution, which was then stirred for 30 min. After 20 min, 5 mg of nanocomposite was added to this solution and swirled. The absorbance at a certain wavelength (665 nm) was then measured by adding a small quantity of this solution to a cuvette every 5 min. The dye inside the nanocomposite had completely broken down after one hour. The % dye degradation was calculated using the equation suggested by Anwar et al.^52^.

1 $${\text{Degradation}}\; \, \left( \% \right) \, \; = \;\left( {1 - \frac{A}{{A^{ \circ } }}} \right)*{1}00$$

### Ethical approval

The collection of plant materials used in this study complies with relevant institutional, national, and international guidelines and legislation.

## Results and discussion

### Optical properties

**Figure 1:**
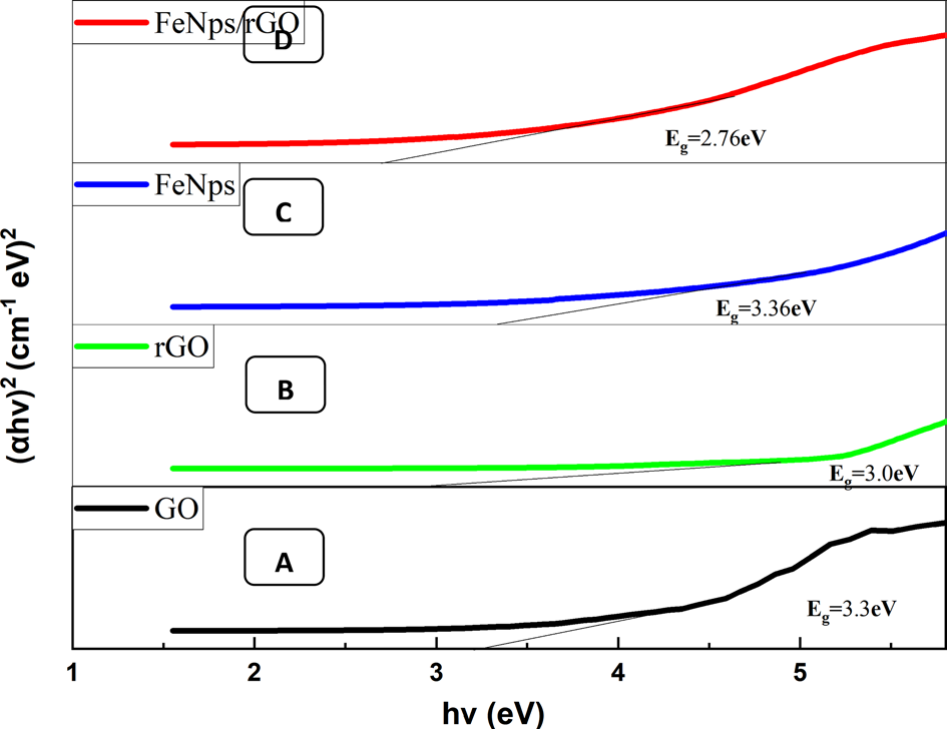
Wood and Tauc plots for (**a**) GO (**b**) rGO, (**c**) FeNPs, and (**d**) FeNPs/rGO nanocomposite using the green deposition method.

For evaluations of optical properties, absorption spectra of synthesized nanomaterials and nanocomposites were analyzed using UV–visible spectroscopy in the 200–800 nm range. The maximum absorbance of synthesized nanomaterials and nanocomposites i.e., GO, rGO, FeNPs, and FeNPs/rGO was observed at 232, 265, 395, and 405 nm, respectively, confirming the synthesis of graphene-based nanocomposites of magnetite iron oxides with reduced graphene oxide. Absorption peaks between 230 and 240 nm are characteristic peaks of GO confirming the synthesis of GO. The obtained 232 nm absorption peak in the case of GO can be ascribed to π–π* transitions of the remaining *sp*^2^ C=C bonds^53,54^ that was shifted to 265 nm in the case of rGO after reduction of GO by green method using *Azadirachta indica* leaf extract. During GO reduction, an increase in π conjugation network shifting of absorption towards longer wavelength region is due to less requirement of energy^55,56^. Similarly, absorption spectra of FeNPs and FeNPs/rGO were observed at 395 and 405 nm, respectively, confirming the synthesis of graphene-based nanoparticles and nanocomposites of magnetite iron oxides with reduced graphene oxide. Band gap values were calculated from UV–visible data and were represented in Fig. 1 using Wood and Tauc plots^57^.

### Fourier transform infrared spectroscopy

**Figure 2:**
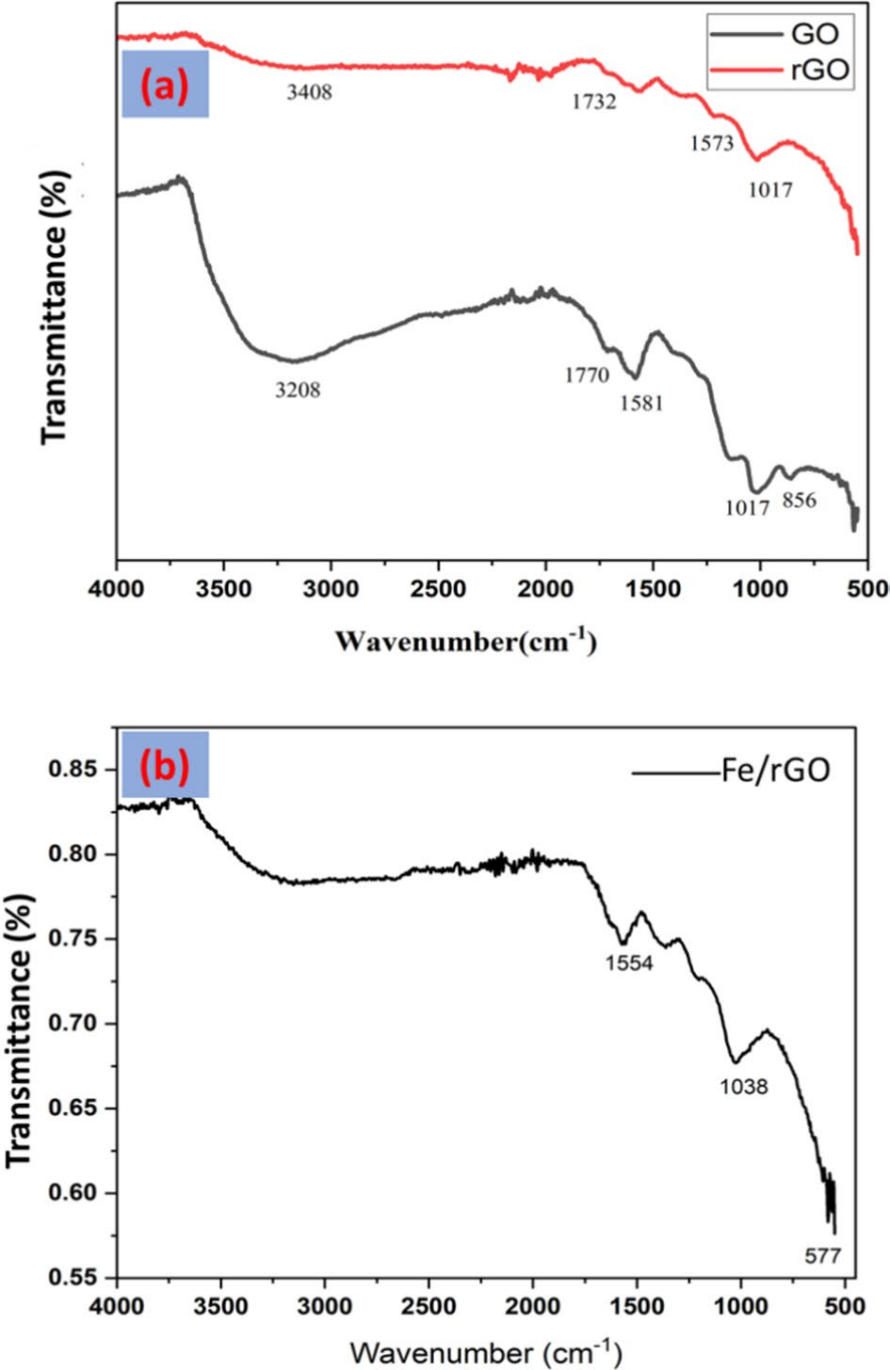
FTIR spectrum of GO synthesized via the chemical method, and rGO synthesized via a green method (**a**), and FTIR spectrum of magnetite iron oxide/reduced graphene oxide (FeNPs/rGO) nanocomposite synthesized by green deposition method (**b**).

Functional groups of GO, rGO, and FeNPs/rGO nanocomposite were observed using FTIR spectrum in the range of 500–4000/cm as shown in Fig. 2a,b. FTIR is a valuable spectroscopic method for characterizing various functional groups, particularly functional groups containing oxygen. The presence of several functional groups including O–H, C–OH, COOH, and C–O in the FTIR spectrum demonstrated that the precursor graphite had been successfully oxidized and GO had been successfully synthesized. A distinctive peak of the stretching mode of O–H functional groups is a large peak of the spectrum between 3500 and 2500 cm^−1^ ^58–60^. The 1573 cm^−1^ peak is attributed to the stretching of the C=C from the unoxidized domain of graphite, whereas the 1730 cm^−1^ peak is attributed to the stretching of the carboxyl group^58,60^. The stretching vibration of C–O from C–O–C is responsible for the 1017 cm^−1^ peak. The reduction of GO into rGO is visible in the FTIR spectrum of rGO, which is depicted in Fig. 2. This is because the O-containing functionalities' peak intensities are less intense than GO's peak intensities. These results demonstrated the reduction of GO caused by ascorbic acid.

The existence of several functional groups in rGO, however, showed that functional groups are still present in the synthesized material despite its close resemblance to pristine graphene which is in agreement with Andrijanto et al.^61^.

**Figure 3:**
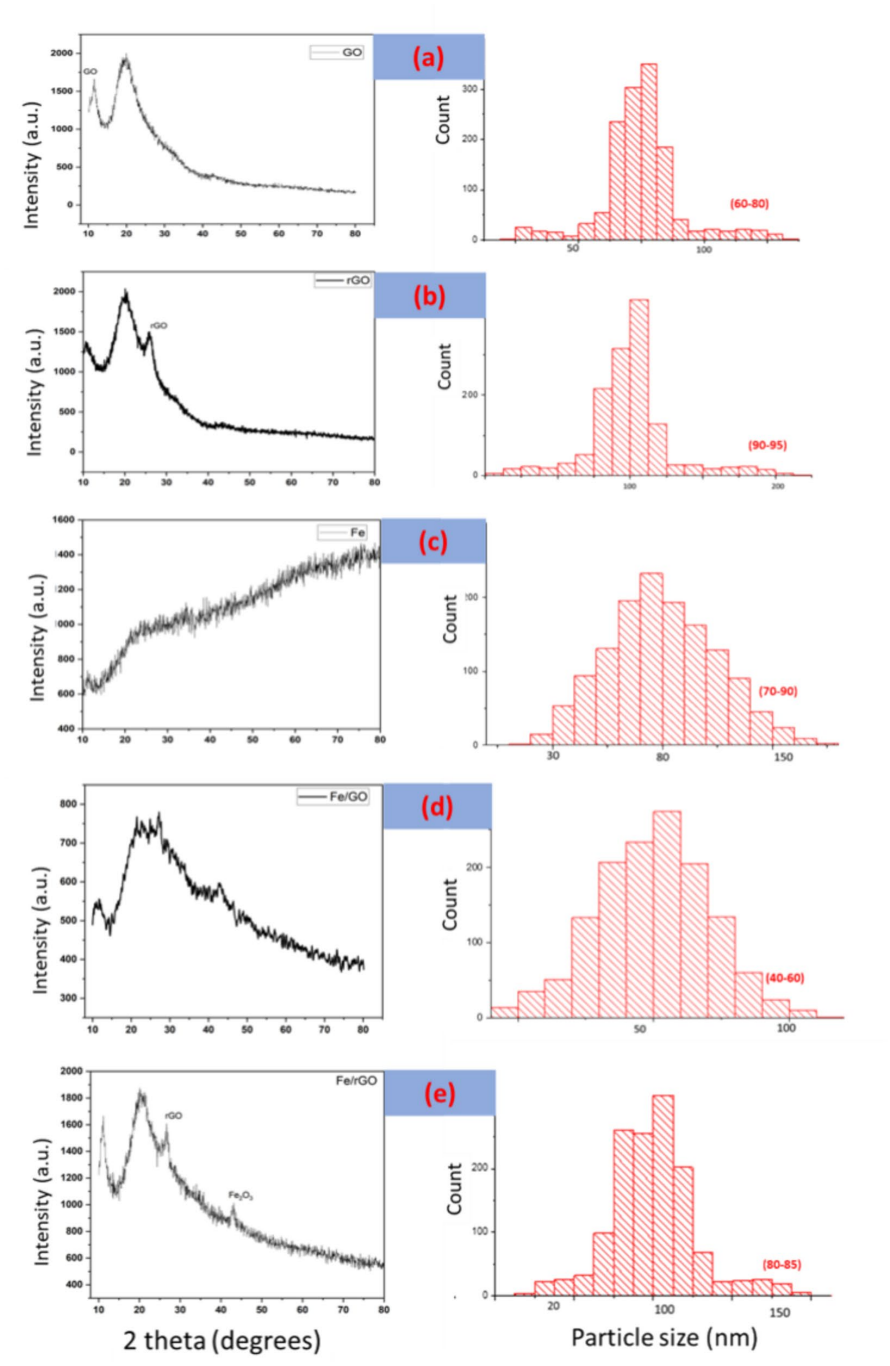
XRD spectrum and mean particle size of (**a**) GO NOs, (**b**) rGO NPs, (**c**) FeNPs, (**d**) FeGO, and (**e**) represents the FerGO nanocomposite synthesized via green deposition method.

FTIR spectrum (Fig. 3) showed a characteristic peak at 577 cm^−1^ which confirmed the synthesis of FeNPs/rGO nanocomposites synthesized by the green deposition method. The absorption band around 577 cm^−1^ was attributed to FeO (indicating Fe_3_O_4_)^62^ and confirmed the synthesis of FeNPs/rGO nanocomposites. These results are in line with those reported by Sodipo et al.^63^.

### X-ray diffraction analysis

The X-ray diffraction pattern of FeNPs/rGO nanocomposites is shown in Fig. 3a–e. The diffraction peak of rGO was about 2*θ* = 26° while the diffraction peak of magnetite iron oxide was about 2*θ* = 44 confirming the synthesis of FeNPs/rGO nanocomposites synthesized via the green deposition method. The diffraction peak of rGO was about 2*θ* = 26° indicating the production of restacked rGO sheets after reduction of GO^64,65^. The JCPDS number for metallic iron nanoparticles, like α-Fe (alpha iron), is often 06–0696, reflecting their crystal structure in X-ray diffraction. However, variations can occur based on factors like nanoparticle size, shape, and surface characteristics. Based on Debye Sherrer's equation, the mean particle size of all synthesized materials was calculated, which follows the standard calculation of nanomaterials. As can be seen in Fig. 3a–e, GO nanoparticles have the particle size of (60–80), rGO nanoparticles are (90–95), FeNPs have the particle size of (70–90); Fe/GO nanocomposites have a particle size (40–60) nm, whereas Fe/rGO nanocomposites are of (80–85) nm. Several studies have examined the synthesis and properties of reduced graphene oxide (rGO) and ferric composites, such as Ma^66^, which presented a controllable rGO/Fe_3_O_4_ composite film. Supriya^67^ investigated the alteration of crystal symmetry in cobalt ferrite-reduced graphene oxide nanocomposites. Studying the synthesis and properties of reduced graphene oxide (rGO) and ferric composites is significant because it can lead to the development of advanced materials with unique properties and applications. These composites have the potential to be used in various fields such as energy storage, catalysis, sensors, and biomedical applications, making them a subject of great interest in scientific research. Sagadevan^68^ presented a chemically stabilized rGO/ZrO2 nanocomposite synthesis with improved electrical properties. Additionally, Singh^69^ discussed the outstanding electromagnetic interference shielding capabilities of a lightweight rGO-Fe3O4 nanoparticle composite. As a result of these studies, rGO and ferric composites have been demonstrated to be effective across a wide range of applications, including magnetoelectronics and electromagnetic shielding.

### Scanning electron microscopy-energy dispersive X-ray analysis

**Figure 4:**
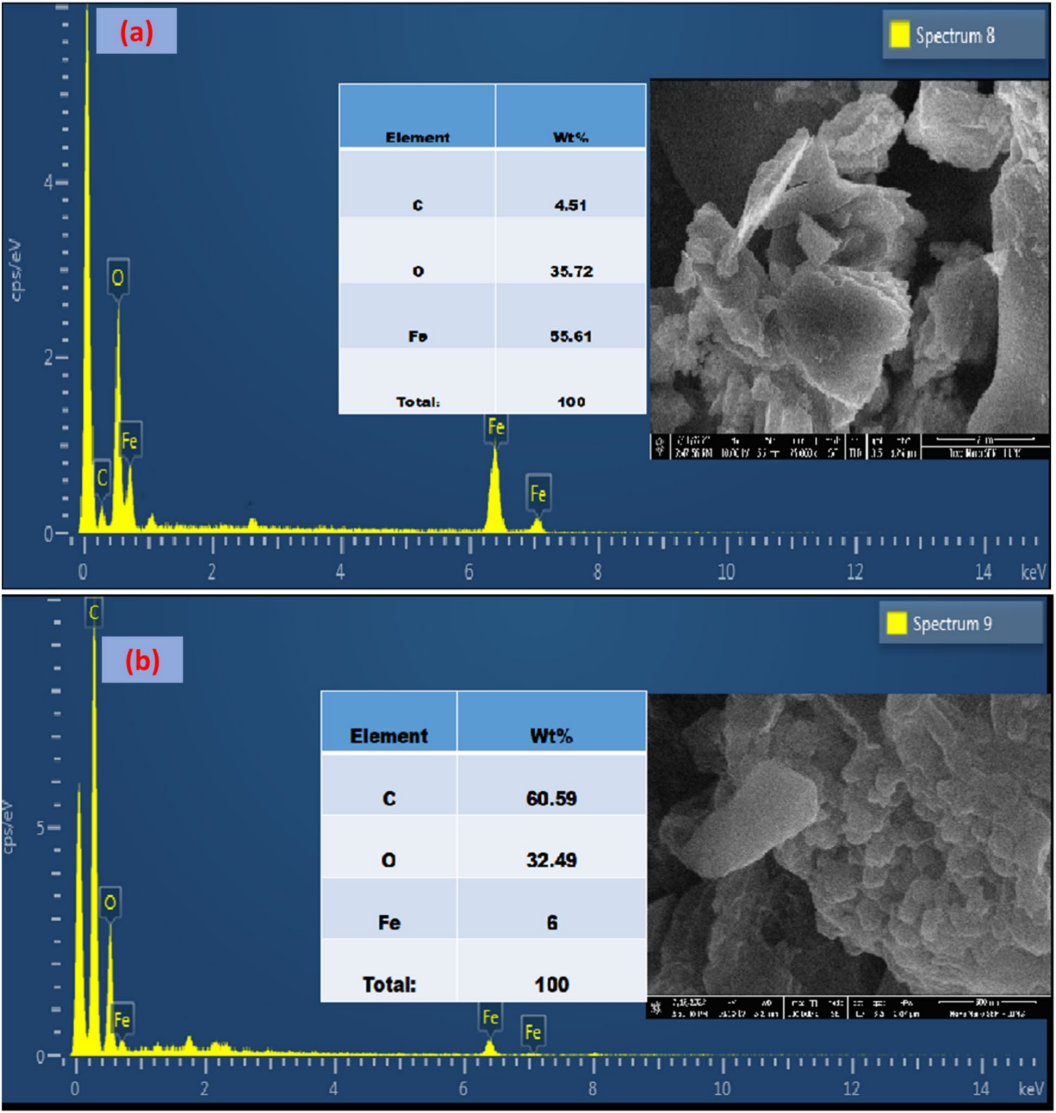
EDX pattern of FeNPs synthesized via green approach (**a**), and EDX graph of FeNPs/rGO nanocomposites synthesized via green deposition method (**b**).

The morphological and elemental distribution of all the synthesized nanoparticles was verified by scanning electron microscopy attached to the energy dispersive spectroscopy examination. Figure 4a,b reveals the EDX elemental mapping of FeNPs and Fe/rGO composites only respectively. It was observed that the percentage of Fe is 55.61%, carbon is 4.51% and oxygen is 35.72% as shown in Fig. 4a. The binding energies of Fe are related to characteristic peaks around 0.9, 6.1, and 7 keV along with the characteristic peak of oxygen at 0.5 keV. Therefore, the EDX analysis confirmed that both iron & oxygen are present in all the nanocomposites. Results are in agreement with those reported by Rahman et al. and Sayed et al.^70,71^.

**Figure 5:**
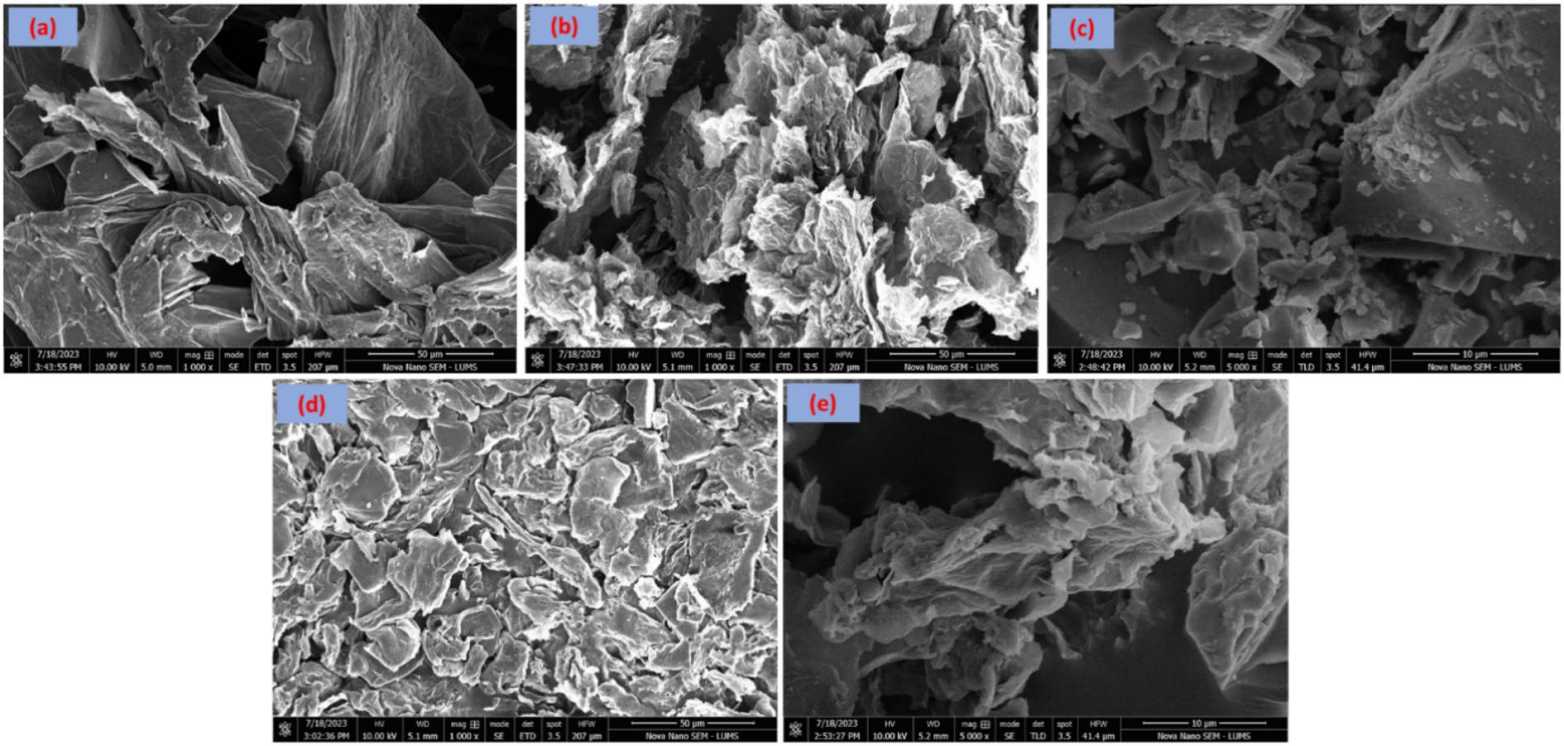
Surface morphology of GO NPs (**a**), rGO NPs (**b**), Fe NPs (**c**), Fe/GO (**d**), and Fe/rGO (**e**) nanocomposites, respectively.

The surface morphology and texture analysis of the synthesized nanocomposites were verified by SEM examination (Fig. 5a–e). Scanning electron microscopy demonstrated the rough surfaces of all the synthesized materials. All types of particles possessed cohesively manifested clustered and spherical shapes. Interactions between graphene-based nanomaterials and magnetite nanoparticles in the nanocomposites are influenced by functional groups and surface chemical properties. Based on the present surface morphologies of the synthesized nanomaterials, it has been established that they can be used for better applications in the removal of dyes and metals from wastewater due to their porous and rough surfaces. These nanomaterials can also be used for the removal of pesticides and other pollutants from contaminated water. These composites of FeNPs are gaining much interest because of their unique optical, electric, and magnetic properties^72^.

### Photocatalytic activity of FeNPs, and Fe/rGO nanocomposite against methylene blue

**Figure 6:**
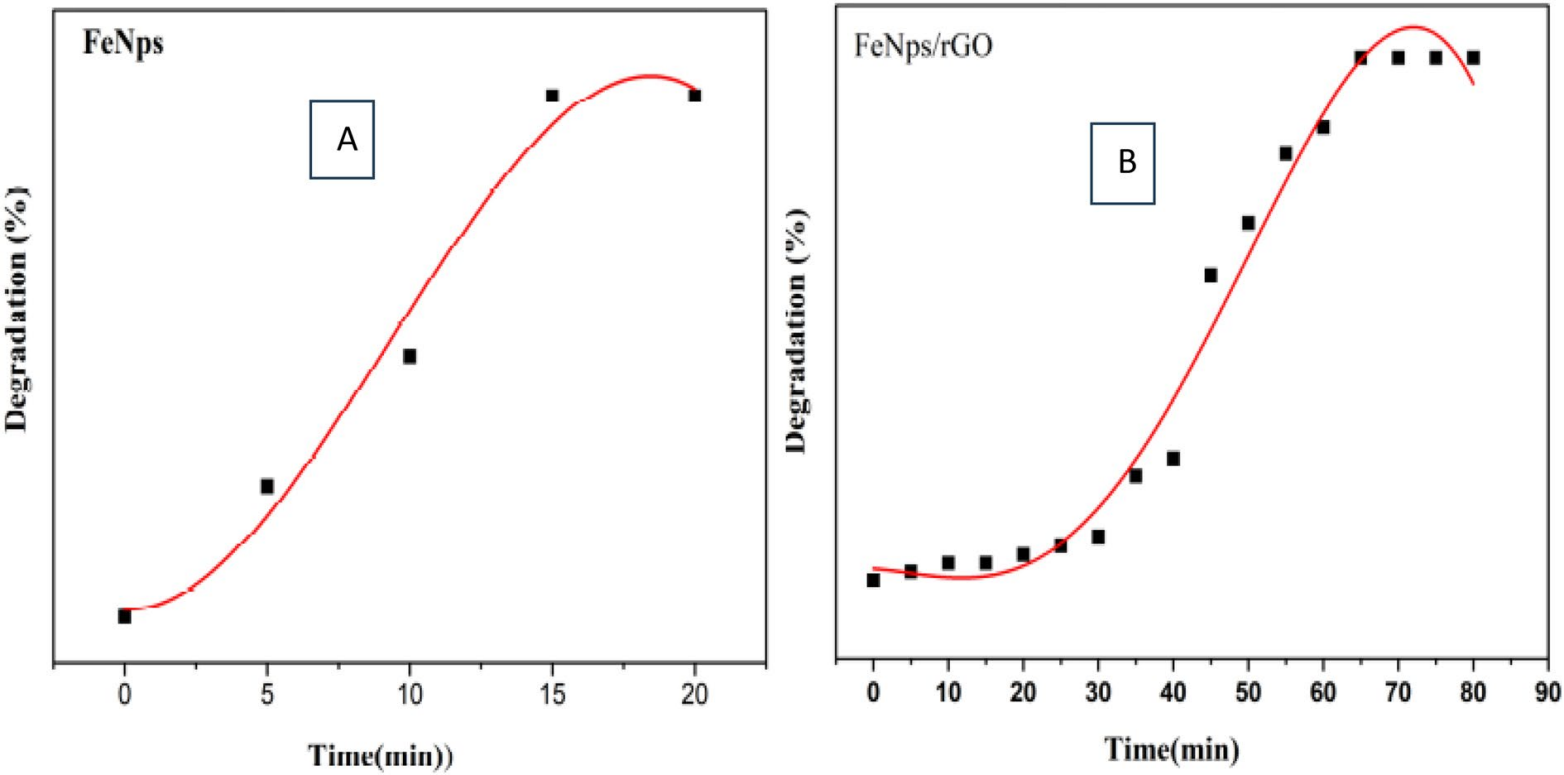
Degradation of methylene blue against time (**a**) FeNPs nanoparticles (**b**) FeNPs/rGO nanocomposites.

**Figure 7:**
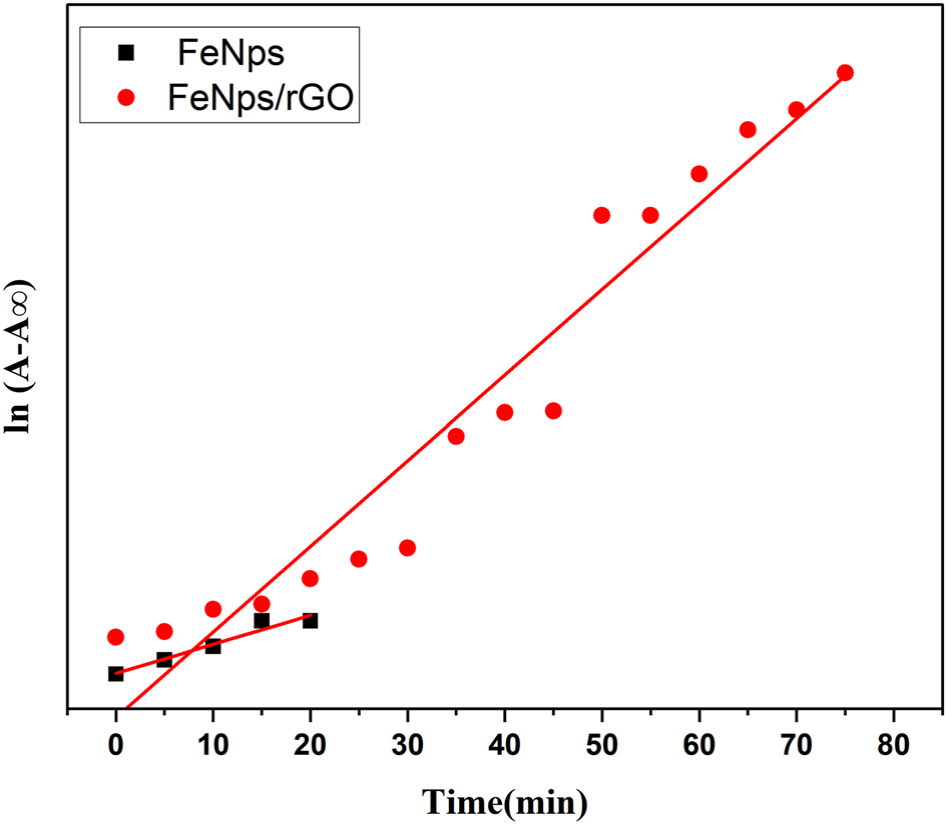
A plot of ln(A − A∞) versus time for rate constant.

The percentage degradation of methylene blue (MB) in samples is represented in Fig. 6a,b. The activity was done with FeNPs nanoparticles and with FeNPs/rGO nanocomposites to estimate their relative performance in the degradation of toxic pollutants. The FeNPs alone showed about 16% degradation (Fig. 6a) while when it was combined with reduced graphene oxide to make FeNPs/rGO nanocomposite, then it showed enhanced photocatalytic activity and degradation was about 75% as shown in Fig. 7b. These results agree with those reported by Abid et al.^73^ and Sadhukhan et al.^74^.

The first order rate Eq. 2 was used to draw the graph between ln(A − A∞) versus time to conduct the kinetic investigation. Figure 7 illustrated this relationship by stating that the slope gave the value of the first order rate constant, k (min^−1^), as

2 $${\text{ln }}\left( {{\text{A}} - {\text{A}}\infty } \right) \, = - {\text{kt}} + {\text{lnA}}\infty$$

The properties and applications of reduced graphene oxide (rGO) and its composites with ferric have been studied in a variety of studies. According to Shahid et al.^75^ and Iftikhar et al.^76^, graphene enhances the photocatalytic properties of rGO composites with different ferric materials. An rGO/Fe_3_O_4_ composite film was examined by Ma et al.^66^, demonstrating its potential for a variety of applications. As a demonstration of the versatility of rGO composites, Deepi et al.^77^ synthesized a novel rGO-SCO nanocomposite with high specific capacitance. The nanocomposite also demonstrated excellent stability in aqueous electrolytes, making it a promising material for energy storage applications. Furthermore, rGO composites have also shown potential for water treatment, corrosion inhibition, and biosensing.

## Conclusions

Graphene-based nanomaterials such as graphene oxide (GO), reduced graphene oxide (rGO), iron nanoparticles (FeNPs), and magnetite iron oxide/G-based nanocomposites (FeNPs/rGO) were successfully synthesized using *Azadirachta indica* leaf extract via green deposition method*.* Synthesized materials were successfully characterized by UV–vis spectrophotometry, Fourier Transform Infra-Red spectroscopy, XRD, and SEM–EDX techniques. The final synthesized FeNPs/rGO nanocomposites showed remarkable photocatalytic activity in the removal of toxic pollutants such as methylene blue dye from the wastewater. FeNPs/rGO nanocomposites showed 75% degradation against methylene blue which was attributed to its lower band gap. Based on these results, it is plausible to conclude that newly synthesized FeNPs/rGO nanocomposites can also be deployed as a potential photocatalyst degrader for photocatalytic degradation of other different dye pollutants due to lower band gap.

## Data Availability

The datasets used and/or analyzed during the current study are available from the corresponding author upon reasonable request.
